# Navigating Diagnostic Uncertainty: Frontal Fibrosing Alopecia Versus Keratosis Pilaris Atrophicans Faciei With Genetic Testing Insights

**DOI:** 10.7759/cureus.58830

**Published:** 2024-04-23

**Authors:** Deesha D Desai, Ambika Nohria, Jerry Shapiro, Kristen I Lo Sicco

**Affiliations:** 1 Department of Dermatology, University of Pittsburgh School of Medicine, Pittsburgh, USA; 2 Ronald O. Perelman Department of Dermatology, New York University (NYU) Grossman School of Medicine, New York, USA; 3 Ronald O. Perelman Department of Dermatology,, New York University (NYU) Grossman School of Medicine, New York, USA

**Keywords:** hair loss, clinical case, genetic testing, keratosis pilaris atrophicans faciei, frontal fibrosing alopecia

## Abstract

Keratosis pilaris atrophicans faciei (KPAF) and frontal fibrosing alopecia (FFA) present diagnostic challenges due to their similar clinical characteristics. Dermatologists often employ overlapping treatment regimens, which may hinder accurate diagnosis and treatment expectations. Genetic testing offers promise for precise diagnosis and tailored treatment strategies, yet its utility in these conditions remains underexplored. This manuscript presents a unique case study of a 36-year-old male with symptoms of both KPAF and FFA, who underwent genetic testing. Despite testing negative for this mutation, the case underscores the potential of genetic testing to enhance diagnostic accuracy and optimize treatment outcomes.

## Introduction

Keratosis pilaris atrophicans faciei (KPAF) and frontal fibrosing alopecia (FFA) share similar clinical characteristics, presenting challenges in diagnosis. Both conditions may manifest features such as bilateral eyebrow loss and facial follicular papules, often accompanied by lymphocytic infiltrates observed in histopathology [1,2]. Dermatologists frequently employ overlapping treatment regimens in an attempt to manage both conditions simultaneously. However, this approach may lack clarity in diagnosis for patients, could result in unnecessary treatments based on the underlying condition, and prohibit providing appropriate treatment expectations. Genetic testing holds promise for precise diagnosis and enables more tailored treatment strategies. Despite this potential, previous studies have not explored cases comparing these conditions in clinical settings or the utility of genetic testing for diagnosis. Herein, we introduce a unique case study of a patient displaying symptoms of both conditions, who underwent genetic testing to uncover insights into the underlying pathology.

## Case presentation

**Figure 1:**
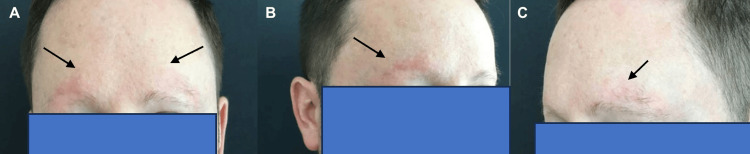
(A-C) Bilateral eyebrow loss with background erythema

**Figure 2:**
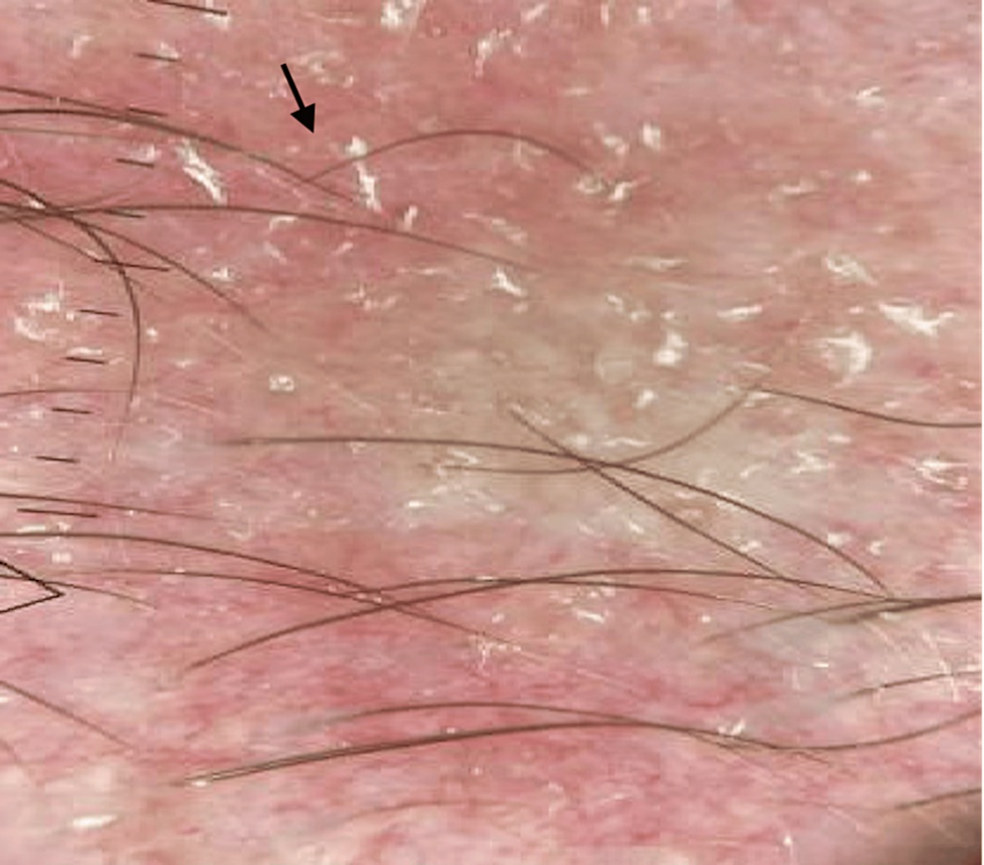
Left lateral eyebrow dermatoscopy demonstrating follicular dropout with background scaling, erythema, and telangiectasias

A 36-year-old male initially sought consultation at the dermatology clinic for a two-year history of hair loss, primarily affecting his eyebrows. The loss had progressed to near-complete absence at the time of consultation. He denied any concurrent loss of scalp hair, eyelash, or beard hair loss, as well as associated symptoms such as itching, pain, burning sensation, or excessive shedding. Despite using topical ruxolitinib 1.5% cream once daily for three months, there was no improvement noted. Physical examination revealed nearly complete bilateral eyebrow loss with dermatoscopic signs of erythema, telangiectasias, and scaling (Figures 1A-1C, 2). A left lateral eyebrow punch biopsy revealed sparse superficial perivascular lymphocytic infiltrate, rare eosinophils, and focal interface changes. Given the suspicion of FFA, Lichen planopilaris (LPP), or KPAF, treatment commenced with medications overlapping for all conditions: tacrolimus 0.1% topical ointment once daily, tretinoin 0.01% gel once daily, and oral minoxidil 2.5mg once daily.

**Figure 3:**
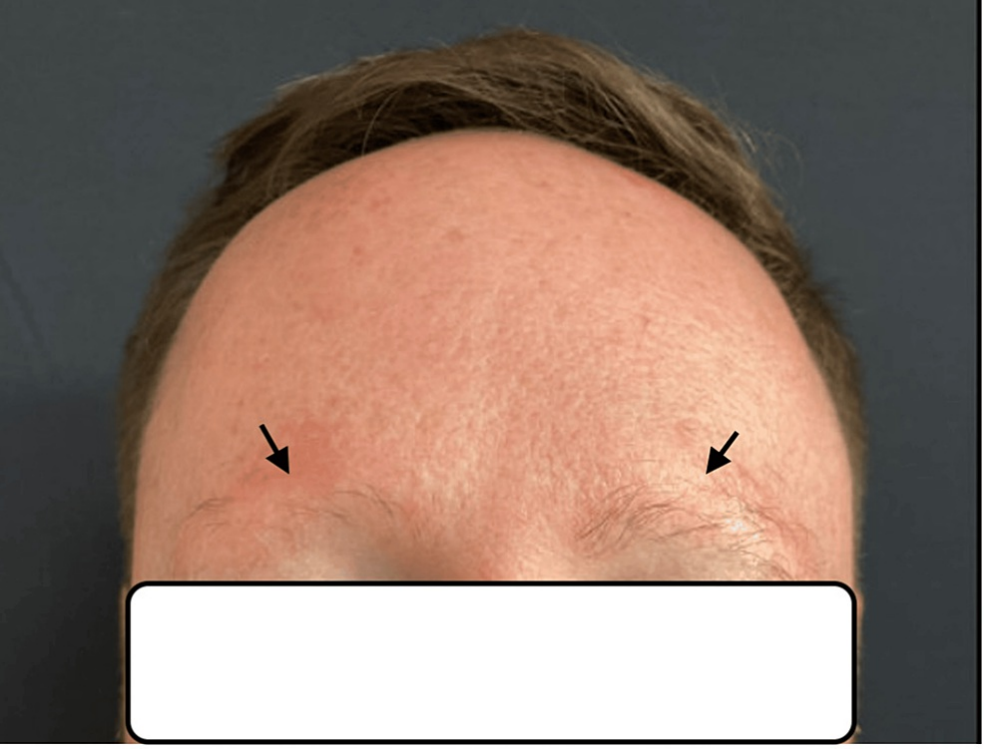
Bilateral eyebrows at the follow-up visit, left > right density

At the follow-up visit six months later, the patient reported mild regrowth of his eyebrows and new-onset irritation at the mid-frontal hairline. Physical examination revealed increased density in the left eyebrow with persistent erythema but no visible scaling (Figure 3). Thin, scattered perifollicular scaling was observed at the frontal hairline, along with flesh-colored forehead papules. Considering these developments, genetic testing was recommended, specifically targeting the Desmoglein - 4 (DSG-4) mutation. However, the patient tested negative for this specific mutation.

## Discussion

KPAF is theorized to stem from aberrant keratinization within the follicular infundibulum [3]. This process results in the accumulation of scale within the follicle, obstructing the hair shaft and inciting inflammation. Over time, this chronic inflammation culminates in permanent atrophy that is highlighted by signs of telangiectasias that can be seen in trichoscopy of the eyebrows [3]. However, both autosomal dominant and recessive inheritance patterns have been described with specific genetic markers, notably DSG-4, implicated [4]. This condition typically manifests in infancy or childhood and is marked by the presence of facial follicular erythematous papules, permanent loss of lateral margins of bilateral eyebrows, and potentially widespread keratosis pilaris on the limbs and trunk.

In contrast, FFA is believed to have an autoimmune etiology, primarily affecting older adults, notably post-menopausal women. FFA shares similar clinical features with KPAF, including bilateral eyebrow loss (madarosis), perifollicular erythema, follicular hyperkeratosis (noted in the frontal hairline rather than eyebrows), and facial follicular flesh-colored papules, but it is differentiated by a receding frontal hairline (Table 1) [5].

**Table 1:** Keratosis pilaris atrophicans faciei versus frontal fibrosing alopecia

	Keratosis Pilaris Atrophicans Faciei	Frontal Fibrosing Alopecia
Cause	Abnormal keratinization of follicular infundibulum and/or autosomal dominant/recessive inheritance described with specific genetic markers	Unknown, though considered autoimmune
Onset	Infancy or childhood	Older adults, more common in women
Clinical Characteristics	Permanent loss of lateral margins of eyebrows, widespread keratosis pilaris on limbs and trunk and facial follicular papules (erythematous)	Receding frontal hairline, loss of eyebrows, perifollicular erythema and hyperkeratosis and facial follicular papules (flesh-colored)
Histopathology	Superficial perivascular lymphocytic infiltrate	Lymphocytic infiltrate around isthmus and infundibulum, replaced by fibrosis
Eyebrow Findings [6,7]	Grouped keratotic follicular papules, perifollicular erythema along eyebrows, absence of follicular ostia, presence of telangiectasias, yellow dots and dystrophic hair	Yellow dots, multiple pinpoint dots, short thin hairs (vellus), black dots, tapered and broken hair and dystrophic hairs
Genetics [8]	*DSG-4*,* LDL receptor-related protein (LRP-1 gene), Membrane-bound transcription factor protease 2 (MBTPS-2 gene)* and Chromosome 18p arm	*6p21.1* (association with HLA-B*07:02 allele), *2p22.2* (variant in CYP1B1), *8q24.22* and *15q2.1*

These two conditions share clinical overlap. Moreover, the histopathological characteristics of KPAF demonstrate superficial perivascular lymphocytic infiltrate and FFA exhibits lymphocytic infiltrate around the isthmus and infundibulum, eventually replaced by fibrosis. The lymphocytic nature of both conditions adds complexity to their differentiation. Given these similarities, the pivotal question revolves around identifying techniques that can effectively guide therapeutic decisions for these complex conditions.

In light of these challenges, exploring techniques such as genetic testing may offer insights into accurate diagnosis and guide therapeutic decisions. Specific genetic mutations, particularly in genes like DSG-4, LDL receptor-related protein (LRP-1 gene), membrane-bound transcription factor protease 2 (MBTPS-2 gene), and chromosome 18p arm, have been implicated in KPAF's underlying pathology [9,10]. Genetic testing can provide diagnostic clarity, preventing unnecessary treatments based on the true underlying condition and facilitating tailored treatment strategies. In this context, we present a case where genetic testing was utilized in patient care in the hopes of underscoring the potential of this approach. Nonetheless, further studies are imperative to navigate therapeutic decisions effectively in the face of these challenging conditions.

## Conclusions

In conclusion, this case study highlights the intricate diagnostic and therapeutic challenges posed by the clinical overlap between KPAF and FFA. Despite their distinct etiologies, both conditions present notable similarities in clinical presentation and histopathological features, often complicating accurate diagnosis and treatment selection. The case serves as a poignant reminder of the imperative to explore innovative diagnostic modalities, such as genetic testing, to potentially unravel the underlying genetic basis and guide tailored therapeutic interventions. While the patient yielded negative for the specific DSG-4 mutation associated with KPAF, the utilization of genetic testing exemplifies a promising avenue for enhancing diagnostic precision and optimizing treatment outcomes in complex dermatological conditions. Nevertheless, further research endeavors are warranted to elucidate the utility and efficacy of genetic testing in navigating therapeutic decisions amidst the intricate clinical landscape of KPAF and FFA.
